# Changes in Lipids and Lipoproteins after Selective LDL Apheresis (7-Year Experience)

**DOI:** 10.1155/2012/976578

**Published:** 2012-01-24

**Authors:** Genovefa Kolovou, Georgios Hatzigeorgiou, Constantinos Mihas, Nikos Gontoras, Panagiotis Litras, Dimitris Devekousos, Panagiota Kontodima, Constantina Sorontila, Helen Bilianou, Sophie Mavrogeni

**Affiliations:** ^1^1st Cardiology Department, Onassis Cardiac Surgery Center, 17674 Athens, Greece; ^2^Internal Medicine Department, General Hospital “G. Papanikolaou”, Kimi, Greece; ^3^Cardiology Department, Tzanio General Hospital, Piraeus, Greece

## Abstract

*Background*. The aim of the study was to investigate the changes in plasma lipids and lipoproteins and the cardiovascular events after selective LDL apheresis. *Methods and Results*. Two pediatric patients with familial hypercholesterolemia aged 11 and 13 years and 19 dyslipidemic adults aged 41 ± 14 years underwent direct adsorption of lipoproteins (DALI) sessions. The mean follow-up period was 47 ± 23 months. The total cholesterol (TC) values before and after treatment were 8.2 ± 2.2 and 3.1 ± 1.6 mmol/l (318 ± 86 and 122 ± 62 mg/dL), respectively. The interval mean of TC was 6.9 ± 1.9 mmol/l (268 ± 75 mg/dL). The LDL cholesterol concentrations before and after treatment were 6.6 ± 2.1 and 1.7 ± 1.1 mmol/l, (256 ± 82 mg/dL and 65 ± 41 mg/dL), respectively. The percentage of acute LDL cholesterol reduction was 75 ± 11%. Cardiovascular events were observed in seven patients. The average annual event rate was 5.51%. *Conclusion*. LDL apheresis is a very important therapeutic tool in managing patients at high risk for premature CAD or with aggressive CAD, despite adequate medical treatment.

## 1. Introduction

The relationship between serum cholesterol and the risk of cardiovascular disease has been firmly established. For more than 2 decades, multiple clinical trials have documented the cardiovascular benefit of lowering low-density lipoprotein (LDL) cholesterol. Familial hypercholesterolemia (FH) is one of the most common genetic disorders, characterized by increased levels of LDL cholesterol, tendons' xanthomas, and premature atherosclerosis [1]. The frequency of heterozygous (hFH) in most populations is 1 in 500 births with exceptions in Christian Lebanese, where is 1 in 170 births, Afrikaner 1 in 70–100 births, and French Canadian ancestries 1 in 200 births [1, 2]. The homozygous (HFH) is very rare (1 in 1000000 births) with symptoms presenting during childhood and early death due to coronary artery disease (CAD). Approximately 22000 individuals in Greece are assumed to be hFH, with the majority of them being undiagnosed. FH can be caused by mutations in LDL receptor gene (LDLR), the apolipoprotein B-100 gene (APOB), and the protein convertase subtilisin/kexin type 9 (PCSK9) [3, 4]. Worldwide, more than 1200 mutations of LDLR gene have been reported [5]. The clinical diagnosis of FH is based on various diagnostic criteria such as Simon Broome, Dutch, and Great Britain [6, 7]. The identification and treatment of affected individuals is very important matter, since it has been documented that there is a significant reduction of morbidity and mortality with the lipid-lowering treatment [8]. Thus, adequate control of plasma LDL cholesterol is crucial. In majority of hFH patients, this can be achieved by diet and drugs therapy combination. However, when the lipid-lowering treatment is inadequate, the LDL apheresis is optional. There are still some considerations about definite indications for LDL apheresis in individuals with hypercholesterolemia. Furthermore, there is a limited published experience about the cardiovascular outcomes of patients treated with LDL apheresis.

Until now, there are three categories of patients considered for LDL apheresis (see National Institute for Health and Clinical Excellence (NICE), clinical guidelines) [9]:

1. HFH whose total cholesterol (TC) remained 9 mmol/l (350 mg/dL) or decreased <50% on drugs therapy,
2. individuals with FH, progressive CAD and TC remained 4.9 mmol/l (190 mg/dL) or decreased by 40% on maximal drugs therapy,
3. exceptionally, individuals with lipoprotein (a) [Lp(a)] > 60 mg/dL and progressive CAD whose LDL cholesterol remained >3 mmol/l (120 mg/dL) despite drug therapy.

Our aim was to present the impact of LDL apheresis in children and adults with TC values resistant to current available hypolipidemic medical treatment.

## 2. Patients and Methods

### 2.1. Patients

Two pediatric patients aged 11 and 13 years old with HFH and 19 adults (mean age 44 ± 11 years) with or without FH were included in the study. Six patients (5 women) refused LDL apheresis treatment, one of whom died few months later. The diagnosis for FH was based on Simon Broome criteria involving DNA analysis (in majority of patients). Patients were treated with low-fat diet and maximum doses of one of the statins (3-hydroxy-3-methylglutaryl coenzyme A reductase inhibitor) such as simvastatin, atorvastatin, rosuvastatin plus ezetimibe and/or colesevelam (since they were available), and/or fenofibrate. The frequency of the procedure (see below) was adjusted individually with an average of 10–15 days. All patients were informed of the risks of the apheretic procedures (allergic reactions, hypotension) before giving written informed consent. This procedure has been applied in Onassis Cardiac Surgery Center since 2004, and the followup was carried out in an open, prospective uncontrolled clinical design.

The diagnosis of hFH was based on the following clinical criteria:

- TC > 7.5 mmol/l (290 mg/dL), LDL cholesterol > 4.9 mmol/l (190 mg/dL),
- presence of tendon xanthomas in the patient or a 1st or 2nd degree relative, are
- history of premature vascular disease in a 1st degree relative <60 years or in 2nd degree relative <50 years old.

In patients with HFH the direct adsorption of lipoproteins (DALI) procedure was offered to those whose TC was remained at 9 mmol/l (350 mg/dL) or decreased <50% on drugs therapy and in patients with hFH, who had progressive CAD and, their TC was remained at 4.9 mmol/l (190 mg/dL) or decreased by <40% on maximal drugs therapy. In patients without FH, the DALI procedure was offered to those, who had exhausted all other options of lowering their TC levels and have an LDL level above 5 mmol/l (200 mg/dL) with CAD or LDL cholesterol level of 7.7 mmol/l (300 mg/dL) without the presence of CAD.

### 2.2. Methods

The main indication for use of the DALI system was inability to reach target LDL cholesterol levels on medical treatment that included diet and cholesterol-lowering drugs.

DALI 500, 750, 1000 (500 + 500), and 1250 (500 + 750) adsorbers were incorporated in the extracorporeal circuit. Dali primer solution, acid citrate dextrose formula A (ACD-A) solution, blood lines, and hemadsorption monitor 4008 ADS (Fresenius HemoCare Adsorber Technology GmbH, St. Wendel, Germany) were used. Prior to the session, the adsorbers were rinsed with 3 × 2000 mL of primer solution at a flow rate of 400 mL/min. The first 2 L contained 20000 IU of heparin. The adsorbers were saturated with citrate during priming.

Prior to the session the patients received an initial intravenous heparin bolus, followed by an ACD-A infusion during the session. ADC-A was first mixed with the patient's blood at a ratio of 1 : 20 and reduced to 1 : 40 after 1500 mL of blood were treated.

Two bilateral vascular accesses by venipuncture, generally in the median cubital veins, were established. At the start of the session, the patient was only connected to the afferent (arterial) line of the extracorporeal circuit. All sessions were carried out under blood pressure and electrocardiogram monitoring, and DALI 750–1250 adsorbers were used for the procedure.

### 2.3. Laboratory Measurements

At the start (before any procedure) and end (just before taking the needle from the afferent arm) of each session, a blood sample was drawn for a lipid profile. Blood was collected in tubes containing EDTA. Plasma TC, triglyceride (TG), and high-density lipoprotein (HDL) cholesterol levels were measured using enzymatic colorimetric methods on a Roche Integra Biochemical analyzer with commercially available kits (Roche). The serum LDL cholesterol levels were calculated using the Friedewald formula [10] only in patients with TG levels <4.5 mmol/l (400 mg/dL). Calculated LDL cholesterol = TC − HDL cholesterol − TG/5.0 (mg/dL). All samples were analyzed within few hours.

The body mass index was calculated as weight divided by height expressed in kg/m^2^.

### 2.4. Statistical Analysis

The results were expressed as mean ± standard deviation (SD). The Student's *t*-test was used to compare the continuous variables between groups, at a significance level of *P* < 0.05.

The acute differences in TGs, HDL cholesterol, and LDL cholesterol before and after LDL apheresis were described as % difference, based on the following rule: % difference = [((variable before-variable after))/variable before] ∗ 100. The correction for haematocrit was made by measuring haematocrit at the start and at the finish of session and multiplying the *C*_min⁡_ value (cholesterol level immediately after an apheresis) by the pre/postapheresis haematocrit ratio.

The interval mean was calculated using the equation devised by Kroon el al. [11] *C*_mean_ = *C*_min⁡_ + *K*(*C*_max⁡_ − *C*_min⁡_). *C*_min⁡_ = cholesterol level immediately after an apheresis and at the start of the subsequent procedure (*C*_max⁡_). Kroon et al. [11] proposed using a value of *K* = 0.73. Because calculation of LDL cholesterol using the Friedewald equation has been shown to underestimate the low concentrations seen immediately after apheresis [12] and we have not performed direct LDL cholesterol, the TC values only were used.

Survival analysis and Kaplan-Meier survival curve were applied in order to evaluate the average annual cardiovascular event rate. Data were analyzed using STATA (Version 9.0, Stata Corporation, College Station, TX, USA).

## 3. Results

The mean follow-up period was 47 ± 23 (range 9–81) months. DALI 750 was performed in 10%, and DALI 1000 in 20% and DALI 1250 in 70%. Twenty-one patients (18 men) underwent 2856 DALI sessions between June 2004 and September 2011. The clinical characteristics of the patients were presented in Table 1. The medication list shows the current medication of participants. Patients who were in angiotensin converting enzyme inhibitors prior entering the DALI sessions were switched to calcium-channel blockers to control the blood pressure. During the follow-up, in two patients, acenocoumarin was added due to aortic valve replacement, and in two patients clopidogrel was added following angioplasty. No other major changes in drug treatment were done.

One patient has been previously treated with one session of plasma exchange. Five patients were diagnosed with HFH and 10 patients with hypercholesterolemia (familial or nonfamilial). Six patients had mixed dyslipidemia with high TC and extremely high TG levels and progression of CAD who did not respond adequately to medical treatment. The average TC, TG, HDL, and LDL cholesterol levels before and after treatment of all and in HFH and non-HFH treated patients was shown in Tables 2 and 3.

The mean acute reduction of all DALI sessions was 75 ± 11% for LDL cholesterol, 50 ± 16% for TG, and 14 ± 7% for HDL cholesterol. The interval mean for TC was 6.9 ± 1.9 mmol/l (268 ± 75 mg/dL).

**Figure 1:**
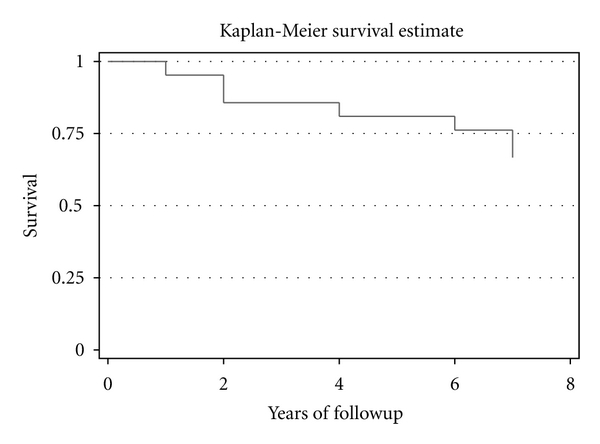
Kaplan-Meier survival curve.

Thirty-three percent of patients experienced cardiovascular events; 2 cardiac deaths (71-year-old women and 54-year-old men) independently of the sessions, 1 coronary bypass grafting, 2 aortic valve replacement, 1 percutaneous coronary intervention angioplasty, and 1 carotid artery angioplasty (pathology report showed scarring of atheromatous plaque and formation of a thick fibrous cap). The average annual event rate was 5.51%, Figure 1.

The major adverse effects (most likely at the first sessions) were allergic reactions manifested as shortness of breath and/or facial flushing, nausea vomiting, serious hypotension, and technical difficulties and were recorded in 10% of the patients. However, minor side effects like puncture difficulties, technical problems, headache, dizziness, or hypotensive periods were present occasionally. All of them were rechallenged, uneventfully.

## 4. Discussion

The DALI system is an exceptional whole-blood cholesterol apheresis system. Since its introduction at the Onassis Cardiac Surgery Center in 2004, 21 patients have been treated. Our results showed 61% acute reduction in TC and 75% in LDL cholesterol beyond that achieved by previous medical treatment. The interval mean for TC was 6.9 ± 1.9 mmol/l (268 ± 75 mg/dL). A reduction of 50% in TGs was also observed. The average annual event rate was 5.51%.

In the LAARS study [13] (angiographic trials), 7 out of 21 patients had cardiac events, 3 unstable angina including one cardiac death, and 4 myocardial infarction over 2 years; however, most of the events took place in the first year. These results were similar to our results. In L-CAPSb [14], 3 out of 25 patients had a cardiac event during a 2.3-year period, and 2 patients underwent coronary intervention within 6 months of enrolment. In the Hokuriku study, where 43 hFH patients with CAD were included [15], patients who were treated with LDL apheresis had LDL cholesterol reduced by 58%. Moreover, during a 6-year follow-up period, coronary events were reduced by 72% (as compared to the group receiving drug therapy alone).

In our group the percentage of event-free survival for 7 years was 67% that was moderately low and close to the control group of the Hokuriku study. Noteworthy to mention, most of the patients, referred to us, were already presented with clinical manifestations of atherosclerosis [16–18]. In the study by Masaki et al. [19], the event-free survival with respect to a major cardiac event (cardiac death and myocardial infarction) was 94.4% for 6 years. The Liposorber Study Group in the United States [20] examined the effects of LDL apheresis in a trial with a 5-year followup of 49 FH patients. The rate of cardiovascular events (cardiac death, coronary revascularization, myocardial infarction, or cerebrovascular events) with LDL apheresis was 3.5 events per 1,000 patient-months of treatment. Keller [21] reported the results of LDL apheresis treatment in HFH, who were followed up for over 30 years. They found 21% deaths in group on LDL apheresis compared with 43% in group without LDL apheresis. In our study in LDL apheresis group, the 9.5% of deaths were reported during a 7-year follow-up period, while, in patients who were not treated with LDL apheresis, the 17% of deaths were reported. Koziolek et al. [22] analysed retrospectively approximately 10000 apheresis sessions performed in 38 patients during the past 20 years (mean of 9 years) in German and showed a decreasing incidence of major coronary events with LDL apheresis.

There is still a small number of studies describing, the 10 year and over, the clinical impact of LDL apheresis and coronary artery disease outcomes. Although these patients are usually at extreme risk to develop major clinical events, several doctors still have some reservation towards referring drug-resistance patients for LDL apheresis. Furthermore, many doctors are not familiar with the regulations concerning the indication reimbursement for LDL apheresis reported by Societies for Apheresis [23–25].

However, the end results are in favor for LDL apheresis that is a promising tool for retarding or arresting the atherosclerosis process in refractory hypercholesterolemic patients. Koga and Iwata [26] in a pathology report showed scarring of atheromatous plaque and formation of a thick fibrous cap after 7 years of LDL apheresis in a FH patient, similar to our finding.

Limitations of the study are the following. The LDL apheresis is an invasive procedure, performed only in selected hospitals on an out- or in-patient basis. During this procedure (which takes approximately 2 hours), an experienced physician and/or technician (nurse) has to be present. Moreover, the procedure still remains expensive.

In conclusion, LDL apheresis is a very important tool in managing patients at high risk of premature CAD, such as those with FH or with progressive, aggressive CAD despite adequate medical treatment. Therefore, the early consideration of LDL apheresis as a lifesaving procedure for these patients is of great value.

## Figures and Tables

**Table 1:** Baseline clinical characteristics of 21 patients with LDL apheresis.

Age, years	41 ± 14
Female	3 (14.3%)
BMI, kg/m_2_	27 ± 4
Hypertension	11 (52%)
Diabetes	4 (19%)
Smoking	5 (24%)

*Previous conditions *	
Myocardial infarction	6 (29%)
Coronary artery bypass grafting	10 (48%)
PCI angioplasty	5 (24%)

*Medications *	
Beta-blockers	12 (57%)
ACE inhibitors	0 (0%)
Nitrates	2 (10%)
Calcium-channel blockers	6 (56%)
Statins	19 (90%)
Fibrates	7 (33%)
Colesevelam	14 (67%)
Ezetimibe	10 (48%)
Aspirin	10 (48%)
Clopidogrel	5 (24%)
Acenocoumarin	2 (10%)
*LDL apheresis period, months *	47 ± 22
LDL cholesterol before entry	9.9 ± 5.9 mmol/l (384 ± 229 mg/dL)

LDL indicates low-density lipoprotein, PCI indicates percutaneous coronary intervention, and ACE indicates angiotensin-converting enzyme. Values were expressed as means ± standard deviation (SD); other values were numbers of patients with percentages given in parentheses.

**Table 2:** Lipid profile before and after LDL apheresis sessions in mg/dL.

	TC	TC HT corrected	TG	HDL cholesterol	LDL cholesterol
Preapheresis	318 ± 86		287 ± 624	41 ± 12	256 ± 82
Postapheresis	122 ± 62	133 ± 68	183 ± 488	35 ± 10	65 ± 41
% difference	−61% ± 13%	−58% ± 14%	−50% ± 16%	−14% ± 7%	−75% ± 11%

All data were expressed as mg/dL, mean ± SD. The mean data were based on multiple measurements (*n* = 1216, only sessions which had at least three parameters, TC, TG, and HDL measured were involved) during the total follow-up period. All values were significantly decreased *P* < 0.001 after the procedure. To convert cholesterol values (TC, HDL, and LDL) from mg/dL to mmol/l, multiply by 0.0259, and, for TGs, multiply by 0.0113. TG indicates triglycerides, LDL indicates low-density lipoprotein, and HDL indicates high-density lipoprotein (HT = hematocrit).

**Table 3:** Lipid profile before and after LDL apheresis sessions in HFH and non-HFH patients.

	TC	TC HT corrected	TG	HDL cholesterol	LDL cholesterol
*HFH*					
Preapheresis	344 ± 81		93 ± 36	37 ± 8	288 ± 81
Postapheresis	111 ± 44	121 ± 47	42 ± 23	32 ± 7	72 ± 45
% difference	−68% ± 8%	−65% ± 9%	−55% ± 14%	−13% ± 7%	−76% ± 10%

*Non-HFH*					
Preapheresis	284 ± 79		541 ± 885	45 ± 14	196 ± 39
Postapheresis	137 ± 78	149 ± 85	367 ± 700	39 ± 12	53 ± 30
% difference	−53% ± 14%	−49% ± 15%	−42% ± 16%	−14% ± 8%	−72% ± 14%

All data were expressed as mg/dL, mean ± SD. The mean data were based on multiple measurements (*n* = 1216, only sessions which had at least three parameters, TC, TG, and HDL, measured were involved) during the total follow-up period. All values were significantly decreased *P* < 0.001, except HDL cholesterol (*P* = 0.265) in non-HFH group. To convert cholesterol values (TC, HDL, and LDL) from mg/dL to mmol/l, multiply by 0.0259, and, for TGs, multiply by 0.0113. TG indicates triglycerides, and LDL indicates low-density lipoprotein, HDL indicates high-density lipoprotein (HT = hematocrit).
